# COMODI: an ontology to characterise differences in versions of computational models in biology

**DOI:** 10.1186/s13326-016-0080-2

**Published:** 2016-07-11

**Authors:** Martin Scharm, Dagmar Waltemath, Pedro Mendes, Olaf Wolkenhauer

**Affiliations:** Department of Systems Biology and Bioinformatics, University of Rostock, Rostock, Germany; School of Computer Science and Manchester Institute of Biotechnology, University of Manchester, Manchester, Great Britain; Center for Quantitative Medicine, School of Medicine, University of Connecticut, Farmington, CT USA; Stellenbosch Institute for Advanced Study (STIAS), Wallenberg Research Centre at Stellenbosch University, Stellenbosch, South Africa

**Keywords:** Ontology, Modelling, Difference detection, SBML, CellML, Version control

## Abstract

**Background:**

Open model repositories provide ready-to-reuse computational models of biological systems. Models within those repositories evolve over time, leading to different model versions. Taken together, the underlying changes reflect a model’s provenance and thus can give valuable insights into the studied biology. Currently, however, changes cannot be semantically interpreted. To improve this situation, we developed an ontology of terms describing changes in models. The ontology can be used by scientists and within software to characterise model updates at the level of single changes. When studying or reusing a model, these annotations help with determining the relevance of a change in a given context.

**Methods:**

We manually studied changes in selected models from BioModels and the Physiome Model Repository. Using the BiVeS tool for difference detection, we then performed an automatic analysis of changes in all models published in these repositories. The resulting set of concepts led us to define candidate terms for the ontology. In a final step, we aggregated and classified these terms and built the first version of the ontology.

**Results:**

We present COMODI, an ontology needed because COmputational MOdels DIffer. It empowers users and software to describe changes in a model on the semantic level. COMODI also enables software to implement user-specific filter options for the display of model changes. Finally, COMODI is a step towards predicting how a change in a model influences the simulation results.

**Conclusion:**

COMODI, coupled with our algorithm for difference detection, ensures the transparency of a model’s evolution, and it enhances the traceability of updates and error corrections. COMODI is encoded in OWL. It is openly available at http://comodi.sems.uni-rostock.de/.

**Electronic supplementary material:**

The online version of this article (doi:10.1186/s13326-016-0080-2) contains supplementary material, which is available to authorized users.

**Electronic supplementary material:**

The online version of this article (doi:10.1186/s13326-016-0080-2) contains supplementary material, which is available to authorized users.

## Background

Modelling plays an important role in the life sciences. The multi-disciplinary approach requires scientists to reuse other work. This task is greatly supported by common languages to describe model-based results [1]. Computational models of biological systems are frequently described in XML standard formats such as the *Systems Biology Markup Language* (SBML, [2]) or CellML [3]. These formats have several advantages: Models can be simulated, analysed, and visualised using different software tools; models encoded in standard formats may outlive the tool used to create the model; model exchange becomes feasible; and models can more easily be shared, published, and reused. SBML and CellML focus on encoding the biological network, the mathematics, and the dynamics of the system. This information enables the technical reuse of model code. However, sustainable model reuse requires a basic understanding of (i) the biological background, (ii) the modelled system, and (iii) possible parametrisations under different conditions. For this purpose, terms from ontologies and controlled vocabularies can be linked to the model, adding a semantic layer.

Bio-ontologies are formal representations for areas of knowledge [4]. They clarify the intended semantics of the data, which makes the data more accessible, sharable, and interoperable [5]. An ontology is a tool to provide meaning to data, the information of which can then be subjected to algorithmic processing [6, 7]. For example, the Gene Ontology [6] provides additional information on the genomic level, the NCBI Taxonomy [8] provides information about the nomenclature of species, and UniProt [9] provides information about proteins. We believe that a similar approach should be taken for the semantic description of differences between versions of a model. Using the semantic layer to describe changes in a model allows for storing meaning together with possible implications of these changes. Changes can then be filtered and analysed automatically.

Models evolve over time. Both before and after publication, regular changes lead to different versions of a model [10]. For example, modellers test different hypotheses, maintainers of databases initially curate the model, and other scientists later on correct or extend it. Specifically, semantic annotations are added, parameter values are updated, errors are corrected, models are adopted to changes in the underlying format, etc. On average, a model changes 4.87 times during the first five years after publishing in an open repository^1^. It is important to track these changes for a number of reasons. Changes in parametrisations or on the underlying network may lead to a situation where the original results are not reproducible anymore. Furthermore, all contributions to the model code should be correctly attributed. With respect to simulation results, change records can help to predict modifications in the simulation outcome. Finally, a good communication of model changes increases the trust of scientists wishing to reuse a model for their own purposes.

In our previous work we developed BiVeS, an algorithm to identify and communicate the changes between two versions of a model [11]. BiVeS encodes changes in an XML file and, thus, tools can visualise and post-process identified changes. Small changes might be easy to grasp without the aid of a principled annotation scheme. However, as the list of changes increases it becomes harder to grasp their relevance. To address this problem, we present an ontology to annotate the changes identified with the BiVeS algorithm. The ontology is sufficiently generic that it can be used to annotate the differences between computational models, including those encoded in SBML and CellML.

## Results

We developed the COMODI ontology, because COmputational MOdels DIffer. COMODI provides terms that describe changes in models. These terms can be linked to single differences between two versions of a model, such as typo corrections in a parameter name. Based on the resulting set of annotations, differences can be characterised and classified.

### Design considerations and identification of terms

COMODI was developed based on a study of changes in versions of SBML and CellML models. The models were retrieved from the respective model repositories. More specifically, we started our investigation by manually analysing a predefined set of cell cycle models^2^ from BioModels [12]. We subsequently extended this set with randomly chosen models from both, BioModels and the Physiome Model Repository [13]. The single steps of development are summarised in Fig. 1 and explained in the following:

1. Using the BiVeS algorithm we identified the differences between all subsequent versions of each model and exported the deltas in XML-encoded files. A delta is a set of operations on entities (nodes or attributes, respectively) necessary to transform one document into another [11].

**Fig. 1:**
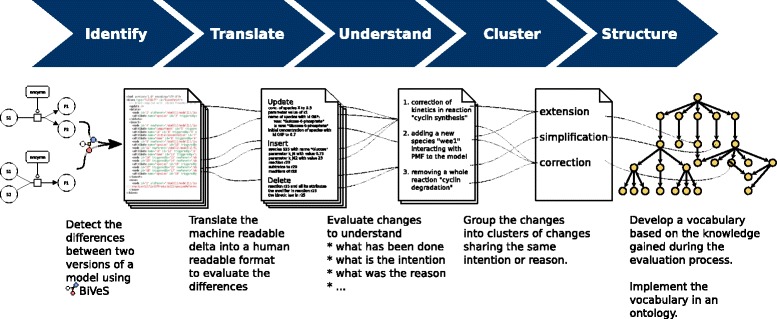
Development process of COMODI. The development process involved five steps with several iterations. First, we used BiVeS to generate the differences between all subsequent model versions. Second, we converted the formal description of more than 10000 differences into human-readable descriptions. Third, we manually studied these descriptions and derived hypotheses and explanations for them. Fourth, we grouped the human-readable descriptions into sets of concepts and derived candidate terms for the ontology. Fifth, we aggregated and classified these terms and implemented the first version of the ontology in Protégé
2. Each found difference was manually translated into a human-readable description and recorded in a wiki software to share and discuss it with collaborators. In total, we investigated more than 10000 differences.
3. Afterwards, we manually analysed the verbose descriptions of changes to understand their effects on the model and to derive hypotheses and explanations for a change. For example, the change of an entity name from *Gulcose* to *Glucose* renames a species and can be considered as the Correction of a Typo that effects an EntityName.
4. We then grouped the changes into several logical clusters, according to the derived hypotheses and explanations of a group of changes. These clusters are based on our own experiences and on feedback from domain experts. The knowledge we gained led to candidate terms for the ontology. We used the human-readable description as a basis for the term definitions.
5. In a last step, we designed a first version of the ontology from the obtained clusters. The ontology was afterwards extended with concepts stemming from standard formats (SBML and CellML terminology, e.g. ParameterSetup) and from the XML domain (e.g. EntityIdentifier). Thus, COMODI contains a whole subtree that specifically focuses on XML encodings.

We quickly identified technically driven properties of changes. For example, it is easy to determine the type of a change as BiVeS already distinguishes between *insertions*, *deletions*, *updates*, and *moves* of entities in XML documents. Moreover, it is always possible to specify the XML entity that is subject to a change. It was, however, more difficult to identify terms describing the reason, intention, or target of a change. The absence of appropriate terms led us to derive new terms from our human readable description of changes. The initial set of terms was then shaped in discussions with other researchers. Throughout the development of COMODI we sought feedback from experts in the fields, e.g., through personal communication or poster presentations at meetings. Finally, we implemented the derived ontology in the Web Ontology Language (OWL) [14] using Protégé [15].

### Ontology organisation and content

**Fig. 2:**
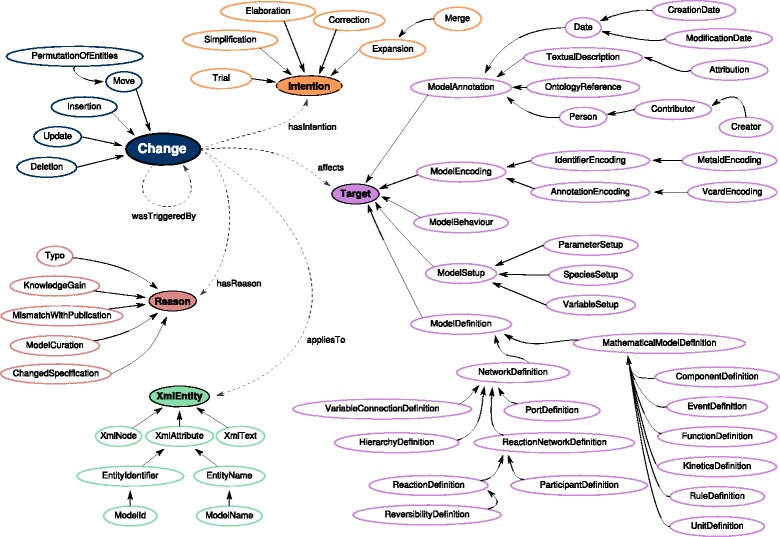
Structure of the COMODI ontology. Differences between computational models can be annotated with the Change term. Using the properties appliesTo, hasIntention, hasReason, and affects, the differences can be linked to the terms of the four major branches of COMODI: XmlEntity, Intention, Reason and Target. All *arrows* between terms within these five branches indicate an is-a relation, unless labelled otherwise

COMODI is organised into four branches around the central concept Change: XmlEntity, Intention, Reason, Target (cf. Fig. 2). As a running example, we use the change of a parameter in an imaginary SBML model. We assume that the parameter changed from 0.5 to 0.8 in the new version of the SBML model.

The subtree rooted by the Change class can be used to specify the type of a change in more detail. Model entities may be inserted (Insertion), deleted (Deletion), updated (Update), or moved (Move). In our example, the modification of the parameter value from 0.5 to 0.8 corresponds to an update (Update) of an attribute value.

Many models are encoded in XML documents. In these cases, a change is always applied to a certain XmlEntity. We distinguish between an XmlNode, an XmlAttribute, or an XmlText element. The update of the parameter value in our example is applied to an XmlAttribute.

Intention and Reason both indicate the purpose of a change. On the one hand, the Intention specifies the aim of a change, particularly with respect to consequences in the future. In our example, the intention of modifying the parameter value is a Correction. On the other hand, a Reason specifically focuses on the cause of a change. In our example, a MismatchWithPublication caused an update of the parameter value.

Most prominent is the Target branch. It contains terms to specify possible targets of a change. COMODI basically distinguishes between five layers in a model document, that can be subject to a change:

1. The ModelEncoding corresponds to the formal encoding of the model document. Terms of this branch can, for example, be used to describe an update of the underlying SBML specification.
2. The ModelAnnotation branch corresponds to the semantic layer of a model document. Terms of this branch can, for example, be used to capture changes in the annotations.
3. The ModelDefinition refers to the actual biological system, for example a reaction network. Terms of this branch can, for example, be used to specify the parts of a model that are affected by a change.
4. The ModelSetup branch can be used to describe changes in the simulation environment. Terms of this branch can, for example, be used to describe changes in parameter values.
5. The ModelBehaviour links to the TEDDY ontology [16]. Thus, it is possible to capture changes in the dynamics of the system. Such changes may, for example, affect the stability characteristics.

Finally, different changes might be linked to each other if they have mutual dependencies. For example, the deletion of a biological reaction triggers the deletion of its kinetic law. Similarly, the deletion of an XML node (e.g. an SBML species) triggers the deletion of all its attributes (e.g. the species’ initial concentration). Those changes can be linked using the wasTriggeredBy relationship to express relations between changes.

COMODI version 2016-03-11 contains a hierarchy of 65 classes and includes five object properties. The object properties can be used to establish relationships between members of the Change class and members of the four main branches of the ontology. We list and explain these properties in Table 1.

**Table 1:** List of object properties defined in COMODI

Name	Description	Domain	Range
affects	Provides information about the parts in a model that were affected by a change.	Change	Target
appliesTo	Stores information about the entity type in an XML document that was changed.	Change	XmlEntity
hasIntention	Links a change to an intention that was to be achieved by the corresponding change.	Change	Intention
hasReason	Links a change to a reason that made this change necessary.	Change	Reason
wasTriggeredBy	Represents dependencies among changes: A change might trigger further changes.	Change	Change

### Availability

The COMODI ontology is licensed under the terms of the Creative Commons Attribution-ShareAlike 4.0 International License^3^. The OWL encoding of the latest version may be downloaded from http://purl.uni-rostock.de/comodi/comodi.owl. Additionally, users may also browse the ontology at http://purl.uni-rostock.de/comodi/. COMODI is also available at http://bioportal.bioontology.org/ontologies/COMODI through BioPortal [17].

## Applications

The COMODI ontology is specifically designed for the annotation of differences between versions of a computational model in the life sciences. In the following we show the usefulness of COMODI for annotating changes, predicting the effects of changes on the simulation result, and filtering versions of a model for specific differences.

### Annotation of changes

SBML models typically use parameters to define the kinetics of a process. The corresponding entity in the SBML document looks as follows:



Here the value of the parameter Km1 is 23.24 molesperlitre.

Updating the parameter value to 23.42 molesperlitre results in an update of the corresponding XML entity. The new version of the model contains the following piece of SBML code:



BiVeS identifies the difference as an update of the paramter value. The XML-encoded serialisation provides the new and the old value of Km1:



Using COMODI the detected update can now be annotated. Some information can directly be inferred and thus be annotated automatically with BiVeS. For example, we know that the above is an update and can link it to the XML entity XmlAttribute. BiVeS is even able to recognize that this change corresponds to a change of the ParameterSetup. The combination of several statements using terms of the different branches allows users to be very specific. COMODI offers terms describing the reason and the intention of a change. Following the example from the previous section, the annotation of the parameter update might look like^4^:



The full example is included in Additional file 1 and explained in Additional file 2.

### Prediction of the possible effects of a change

The modification of the ParameterSetup also affects the ModelSetup (cmp. ontology terms in Fig. 2) and thus potentially influences the simulation results. Similarly, modifications of a FunctionDefinition or the KineticsDefinition can influence the simulation outcome. Finally, changes in the network structure (e. g., modification of the ReactionNetworkDefinition by transforming a reactant into a modifier) will not only affect the simulation outcome, but in addition the visual representation of the network. For this subset of changes, modellers should be notified of any modification.

Another case are changes that affect the ModelEncoding. For example, models are regularly updated to remain compliant with new versions of format specifications. These changes are especially relevant for software tools dealing with model code. As not all tools feature the full set of SBML constructs [18], for example, the upgrade of a model may require the use of another software tool. Thus, changes that result from modifications of the format specification can be of indirect interest for modellers. They may not affect the modelled system but the tools that are needed to interpret and simulate it.

However, other changes may not be as relevant. It can be helpful to hide them and thereby help users focus on important changes. For example, the reading and subsequent writing of a model file using different software tools, such as COPASI [19] or CellDesigner [20], often results in a re-shuffling of elements within the document. However, the sequence of certain elements might not matter to the encoded model. In SBML for example, the order of parameters defined in the listOfParameters is irrelevant for the encoded system, as SBML does not give any semantic meaning to element orders [21]. Thus, changes that only affect the order of elements, can be neglected. Even if BiVeS reports them in its XML serialisation, these changes can be discarded if annotated with the corresponding COMODI terms. For other types of changes, the decision whether to neglect a change or not depends on the user. A new identifier scheme for the semantic annotations, for example, is relevant to curators and tool developers, while it is probably irrelevant for the majority of modellers. However, modellers who based their model analysis, comparison, or visualisation on semantic annotations need to be notified about this type of change. Here, COMODI terms need to be evaluated based on the users’ preferences.

### Filtering by change

The COMODI ontology enables software to automatically filter the list of changes to show only the relevant changes for a given question. For example, if a developer is interested in the model versions before and after an update of the SBML specification, he or she can search for changes annotated with ChangedSpecification and display only those versions of a model that are linked to such a change.

Another, more complex filter is the one for “relevant changes only”. It is difficult to determine what exactly a relevant change may be, and relevance depends on the application domain and user. However, in the context of curation, the curators could define their set of changes that they want displayed and neglected, respectively. The needs of specific user groups may result in different profiles.

Filtering can also be used to display only model versions that are the result of a specific change, while neglecting all other versions. For example, each release of BioModels generates new model versions. However, if the only changes are updates of the SBML level, for example, then it suffices to display a reduced number of model versions to the user, instead of providing them all released versions.

## Conclusions

COMODI is an ontology to describe the differences between two versions of a computational model. The ontology terms specify the type of change for each detected difference. Usually, a combination of COMODI terms from different branches is necessary to characterise a change sufficiently. We currently use COMODI for the description of changes between two versions of the same model, either encoded in SBML or in CellML. However, COMODI terms can also be applied to differences detected between models in any other encoding format, including even code from proprietary languages such as MATLAB.

The COMODI ontology was developed based on a manual study of changes in versions of curated SBML models from BioModels and in versions of CellML models from the Physiome Model Repository. These models, however, were all implemented in core SBML and core CellML. That means, we did not consider extensions. In the future, the study should be extended to also cover SBML models that use SBML extension packages. However, to date no sufficiently large test data exists to derive ontology terms with the workflow described in Fig. 1.

Since version 1.7, the BiVeS tool^5^ supports annotations of detected differences with COMODI terms. BiVeS automatically generates annotations from two branches, ie., XmlEntity and Target. These annotations can be stored together with the differences inside BiVeS’ XML serialisation. However, we suggest that annotations are stored independently to reduce the size of BiVeS’ diff files. For example, the BiVeS file and the annotation file could easily be assembled together with other model-related data in a COMBINE archive [22] or in a Research Object [23, 24]. This way, the annotations can be evaluated, and the difference file does not become unreasonably bloated.

The COMODI ontology encodes knowledge exclusively on the model level. Already now, tools such as SEEK [25], JWS [26], or COPASI [19] can benefit from storing and evaluating information about model changes using COMODI. The ontology is also useful for recording the history of a model, and ensures better transparency of a model’s evolution. Furthermore, it enhances the traceability of updates and error corrections in existing models.

COMODI cannot, however, be used to encode provenance, such as information about the user who changed the model or information about the tool used to update the file. It can, however, easily be coupled with ontologies for provenance. Specifically, PROV [27] and PAV [28] offer some compelling concepts for model provenance, which modeling tools and platforms should take the responsibility of implementing support for. For example the developers of COPASI are currently implementing mechanisms to allow users to easily keep a record of model versions. Each version will be documented by the modeler with free text comments but these are in natural language and therefore not easily machine-readable. To allow for machine-readable annotations the software will also facilitate users to specify COMODI terms as version annotations. Additionally to tracking versions, which are user-definable, COPASI will also track a full provenance log for each model; this is a complete history of the model changes recorded automatically as they happen and serialized in a machine-readable XML format. COMODI terms will then be particularly useful to annotate all the changes as they happen.

We actively support the inclusion of new terms resulting from changes in SBML package files. We are also curious to learn how COMODI can be useful to characterise changes in logical models, large metabolic networks, neuroscientific models etc. We would therefore like to encourage the community to provide further terms for COMODI. We constantly seek expertise and input from developers, modellers, and other scientists. Please submit feedback and requests for additional terms at the github project^6^.

## Endnotes

^1^most.sems.uni-rostock.de/, retrieved May 2nd, 2016

^2^ 108 versions of the models with ids BIOMD0000000005, BIOMD0000000006, BIOMD0000000007, BIOMD0000000 056, and BIOMD0000000107

^3^http://creativecommons.org/licenses/by-sa/4.0/creativecommons.org/licenses/by-sa/4.0/

^4^ This annotation is serialised using the TURTLE format, see http://www.w3.org/TR/turtle/w3.org/TR/turtle

^5^https://sems.uni-rostock.de/projects/bives/sems.uni-rostock.de/projects/bives/

^6^https://github.com/SemsProject/COMODI/wiki/ Please-Send-Commentsgithub.com/SemsProject/COMODI/wiki/Please- https://github.com/SemsProject/COMODI/wiki/Please-Send-CommentsSend-Comments

## Additional files

Additional file 1 Example combine archive. (OMEX 4 kb)

Additional file 2 Explanation for the ExampleArchive.omex. (PDF 190 kb)
