# Supplementary material for: COMODI: an ontology to characterise differences in versions of computational models in biology
Source: J Biomed Semantics. 2016 Jul 11;7:46. doi: 10.1186/s13326-016-0080-2 (PMC4940905; doi:10.1186/s13326-016-0080-2)
Supplement: Additional file 2 — Explanation for the ExampleArchive.omex. (PDF 190 kb) [file 13326_2016_80_MOESM2_ESM.pdf]

Supplementary Information

COMODI: An ontology to characterise differences in versions of computational models in biology

Martin Scharm [1], Dagmar Waltemath [1], Pedro Mendes[2,3], and Olaf Wolkenhauer [1,4]

[1]Department of Systems Biology and Bioinformatics, University of Rostock, Rostock, Germany

[2]School of Computer Science and Manchester Institute of Biotechnology, University of Manchester, Manchester, Great Britain.

[3]Center for Quantitative Medicine, School of Medicine, University of Connecticut, Farmington, CT, USA.

[4]Stellenbosch Institute for Advanced Study, Wallenberg Research Centre at Stellenbosch University, Stellenbosch, South Africa

The COMODI ontology is specifically designed for the annotation of differences between computational models in the life sciences. This example shows how COMODI terms can be used to annotate the differences between two SBML files. The files described in this example are attached to this document as a COMBINE Archive (BAM+14). For more information on the COMBINE Archive format, please refer to (BAM+14) or to the description on the COMBINE Archive web page (co.mbine.org/standards/omex).
You may use the COMBINE Archive Web interface (SWP+14) to extract and explore the files described in this archive. To do so, just upload the attached `ExampleArchive.omex` file to webcat.sems.uni-rostock.de.

The Example COMBINE Archive

The attached `ExampleArchive.omex` is a COMBINE Archive containing the following files:

- `version1.sbml`: The original version of our toy model: One parameter and a unit definition.
- `version2.sbml`: The modified version of the toy model: The value of the parameter was updated.
- `differences.xml`: The differences between both versions of the toy model as identified and exported by the BiVeS tool (SWW15).
- `differences-annotated.xml`: The patch file including annotations using terms from COMODI.
- `annotations.rdf`: The externalised annotations in RDF/XML.

The contents of these files are described in the following.

The Model in Two Versions

The file `version1.sbml` encodes for an artificially created SBML model in Level 2 Version 4. The model defines a parameter `Km1` with a value of *23.24 moles per litre*.

```
<?xml version="1.0" encoding="UTF-8"?>
<sbml xmlns="http://www.sbml.org/sbml/level2/version4" level="2" version="4">
  <model name="TestModel_for_IB2013">
    <listOfUnitDefinitions>
      <unitDefinition id="molesperlitre">
        <listOfUnits>
          <unit kind="mole"/>
          <unit kind="litre" exponent="-1"/>
        </listOfUnits>
      </unitDefinition>
    </listOfUnitDefinitions>
    <listOfParameters>
      <parameter id="Km1" name="Km1" value="23.24" units="molesperlitre" />
    </listOfParameters>
  </model>
</sbml>
```

The file `version2.sbml` is a modified version of the first SBML model. It also contains the paramter `Km1`. However, the value of this parameter has been updated to *23.42 moles per litre*.

```
<?xml version="1.0" encoding="UTF-8"?>
<sbml xmlns="http://www.sbml.org/sbml/level2/version4" level="2" version="4">
  <model name="TestModel_for_IB2013">
    <listOfUnitDefinitions>
      <unitDefinition id="molesperlitre">
        <listOfUnits>
          <unit kind="mole"/>
          <unit kind="litre" exponent="-1"/>
        </listOfUnits>
      </unitDefinition>
    </listOfUnitDefinitions>
    <listOfParameters>
      <parameter id="Km1" name="Km1" value="23.42" units="molesperlitre" />
    </listOfParameters>
  </model>
</sbml>
```

Identifying the Differences

We used BiVeS (SWW15) to identify and formally encode the differences between the two versions of a model. The file `differences.xml` is an export from the BiVeS tool, and it encodes the differences between `version1.sbml` and `version2.sbml`. BiVeS identifies zero deletions, zero inserts and zero moves. However, it detects the update of a parameter value (for the first parameter in the file, which is the only parameter in this case, `Km1`).

```xml
<?xml version="1.0" encoding="UTF-8"?>
<bives type="fullDiff" id="bivesPatch">
  <!--BiVeS compiled with: [BiVeS FrameWork v1.9] [BiVeS Core v1.7.1] [BiVeS SBML v1.7.3] [BiVeS CellML v1.6.4] -->
  <update>
    <attribute name="value" id="1" oldValue="23.24" newValue="23.42"
      oldPath="/sbml[1]/model[1]/listOfParameters[1]/parameter[1]"
      newPath="/sbml[1]/model[1]/listOfParameters[1]/parameter[1]" />
  </update>
  <delete />
  <insert />
  <move />
</bives>
```

Annotating the Differences using COMODI

Since version 1.7, the BiVeS tool is also able to annotate identified differences with terms from COMODI. Using the RDF framework, BiVeS exports these annotations either as a separate file or included in the XML patch. The following listing shows the contents of the `differences-annotated.xml` file, which contains the patch including annotations.

```xml
<?xml version="1.0" encoding="UTF-8"?>
<bives type="fullDiff" id="bivesPatch">
  <!--BiVeS compiled with: [BiVeS FrameWork v1.9] [BiVeS Core v1.7.1] [BiVeS SBML v1.7.3] [BiVeS CellML v1.6.4] -->
  <update>
    <attribute name="value" id="1" oldValue="23.24" newValue="23.42" oldPath="/sbml[1]/model[1]/listOfParameters[1]/parameter[1]" newPath="/sbml[1]/model[1]/listOfParameters[1]/parameter[1]" />
  </update>
  <delete />
  <insert />
  <move />
  <rdf:RDF xmlns:rdf="http://www.w3.org/1999/02/22-rdf-syntax-ns#" xmlns:comodi="http://purl.uni-rostock.de/comodi/comodi#" xmlns:foaf="http://xmlns.com/foaf/0.1/" xmlns:ore="http://www.openarchives.org/ore/terms/" xmlns:pav="http://purl.org/pav/" xmlns:prov="http://www.w3.org/ns/prov#" xmlns:rdfs="http://www.w3.org/2000/01/rdf-schema#">
    <prov:Activity rdf:about="#createPatch">
      <prov:generated>
        <ore:Aggregation rdf:about="#bivesPatch">
          <ore:aggregates>
            <comodi:Update rdf:about="#1">
              <comodi:affects rdf:resource="http://purl.uni-rostock.de/comodi/comodi#ParameterSetup" />
              <comodi:appliesTo rdf:resource="http://purl.uni-rostock.de/comodi/comodi#XmlAttribute" />
```

```
              <comodi:hasIntention
                ↪  rdf:resource="http://purl.uni-rostock.de/comodi/comodi#Correction"/>
              <comodi:hasReason
                ↪  rdf:resource="http://purl.uni-rostock.de/comodi/comodi#MismatchWithPublication"/>
            </comodi:Update>
          </ore:aggregates>
          <rdf:type rdf:resource="http://www.w3.org/ns/prov#Entity" />
        </ore:Aggregation>
      </prov:generated>
      <prov:wasAssociatedWith>
        <prov:SoftwareAgent rdf:about="#bives">
          <pav:version>BiVeS compiled with: [BiVeS FrameWork v1.9] [BiVeS Core
            ↪  v1.7.1] [BiVeS SBML v1.7.3] [BiVeS CellML v1.6.4]</pav:version>
          <rdfs:label>BiVeS</rdfs:label>
        </prov:SoftwareAgent>
      </prov:wasAssociatedWith>
    </prov:Activity>
  </rdf:RDF>
</bives>
```

The example also showcases how PROV (MG13) can be useful when capturing the evolution of a model file. Please note that BiVeS is not able to detect intentions and reasons for a change automatically. We therefore completed the example annotations by manually adding annotations with terms from COMODI.
Please note that the annotations increase the size of the patch file tremendously. We therefore recommend to externalise the annotations and store them in a separate RDF file.

References

[BAM+14] Frank Bergmann, Richard Adams, Stuart Moodie, Jonathan Cooper, Mihai Glont, Martin Golebiewski, et al. COMBINE archive and OMEX format: one file to share all information to reproduce a modeling project. *BMC Bioinformatics*, 15(1):369, 2014.

[MG13] Luc Moreau and Paul Groth. *Provenance: An Introduction to PROV (Synthesis Lectures on the Semantic Web: Theory and Technology)*. Morgan and Claypool Publishers, California, USA, 9 2013.

[SWP+14] Martin Scharm, Florian Wendland, Martin Peters, Markus Wolfien, Tom Theile, and Dagmar Waltemath. The CombineArchiveWeb application – A web-based tool to handle files associated with modelling results. In *Proceedings of the 7th International Workshop on Semantic Web Applications and Tools for Life Sciences, Berlin, Germany, December 9-11, 2014.*, 2014.

[SWW15] Martin Scharm, Olaf Wolkenhauer, and Dagmar Waltemath. An algorithm to detect and communicate the differences in computational models describing biological systems. *Bioinformatics*, 32(4):563–570, 2015.
